# Case Report: Newborns With Pseudohypoaldosteronism Secondary to Excessive Gastrointestinal Losses Through High Output Stoma

**DOI:** 10.3389/fped.2021.773246

**Published:** 2021-11-17

**Authors:** Chia-Yu Ou, Yen-Ju Chen, Geng-Bai Lin, Mei-Fan Chen, Shu-Ti Chia

**Affiliations:** ^1^Department of Surgery, National Cheng Kung University Hospital, College of Medicine, National Cheng-Kung University, Tainan, Taiwan; ^2^Department of Pediatrics, National Cheng Kung University Hospital, College of Medicine, National Cheng-Kung University, Tainan, Taiwan; ^3^Division of Surgical Intensive Care, Department of Surgery, Ditmanson Medical Foundation Chia-Yi Christian Hospital, Chiayi City, Taiwan; ^4^Division of Pediatric Surgery, Department of Pediatrics, Ditmanson Medical Foundation Chia-Yi Christian Hospital, Chiayi City, Taiwan; ^5^Division of Pediatric Surgery, Department of Surgery, National Cheng Kung University Hospital, College of Medicine, National Cheng-Kung University, Tainan, Taiwan

**Keywords:** high output stoma, hyperkalemia, hyponatremia, metabolic acidosis, pseudohypoaldosteronism

## Abstract

Life-threatening electrolyte imbalance is not uncommon in preemies. Differential diagnosis is important for immediate treatment. The syndrome of pseudohypoaldosteronism (PHA) is characterized by increased aldosterone secretion associated with clinical signs of hypoaldosteronism reflecting mineralocorticoid resistance. There are type I, type II, and secondary type of PHA. Most secondary PHA reported in the pediatric population result from urinary infection and obstructive uropathy and extremely rarely from gastrointestinal fluid loss. Seven preemies accepted jejunostomy or ileostomy, and they suffered from high output stoma. Electrolyte imbalance with bodyweight loss or cardiac event was noted. We found a high level of aldosterone and renin and diagnosed them with secondary PHA due to excessive gastrointestinal losses. After stomal reversal, aldosterone and renin level became normalized, and electrolyte was corrected. This study reports the finding of secondary pseudohyperaldosteronism (hyponatremia, hyperkalemia, and metabolic acidosis) in a series of cases with intestinal resection and ostomy of different causes. Early stomal reversal was recommended.

## Introduction

The syndrome of pseudohypoaldosteronism (PHA) is characterized by increased aldosterone secretion associated with clinical signs of hypoaldosteronism reflecting mineralocorticoid resistance (1). Patients with PHA manifested dehydration, hyponatremia, hyperkalemia, and metabolic acidosis. There are type I, type II, and secondary type of PHA (2). Most secondary PHA reported in the infant population resulted from urinary tract infection, obstructive uropathy, and extremely rare gastrointestinal fluid loss. A few adult cases of secondary PHA caused by ileum resection have been reported in the literature, but none of the newborns have reported ileum resection-induced PHA (3–5). The aim of this study is to present our experience in treating newborns with high output stoma-related PHA.

## Case Presentation

Seven patients were enrolled on this case series (Table 1). In our series high output stoma was defined as above 30 cc/kg/day. The gestational age and birth weight of the patients ranged from 23-week preemies to term baby and 560 to 3,780 g, respectively. In case 1, he received ileostomy due to Hirschsprung disease and developed ileostomy diarrhea (>100 cc/kg/day) after gradually increasing enteral feeding. On postnatal day 30, dehydration, hyponatremia, metabolic acidosis, and hyperkalemia were noted. Instead of high output stoma-related hypokalemia, we suspected PHA and checked the aldosterone level. We found serum aldosterone was high (>1,630 ng/dl). Then patient decreased oral intake and accepted the modified Kimura procedure with the right side colon patch fused with ileostomy limb. After ileostomy diarrhea improved, and serum aldosterone level became normal. After this case, we routinely checked serum aldosterone levels in all patients with high output syndrome.

**Table 1:** Demographic data profile of the patients.

**Case**	**1**	**2**	**3**	**4**	**5**	**6**	**7**
Gestational age (weeks)	38	39+4	28+4	24+4	24+5	30+1	23+6
Birth body weight (g)	3,100	3,780	560	616	730	655	653
Gender	M	F	F	M	M	M	M
Disease	Hirschsprung disease	Type 4 intestinal atresia	Necrotizing enterocolitis	Necrotizing enterocolitis with enterocutaneous fistula	Necrotizing enterocolitis	Meconium ileus	Necrotizing enterocolitis
Operation technique	End ileostomy and biopsy	Primary anastomosis, Enterolysis, loop ileostomy	Loop ileostomy	Jejunostomy, STEP bowel lengthening procedure	Loop ileostomy	Loop ileostomy	Loop ileostomy
Stoma location	Ileo-jejunum	Mid-ileum	Mid-ileum	Proximal jejunum	Proximal ileum	Mid-ileum	Proximal ileum
Stoma creation day (Postnatal day)	3	41	4	44	4	8	7
Onset time (Postnatal day)	30	62	39	89	62	38	62
Enteral feeding (cc/kg/day)	192	130	160	130	100	170	110
Stoma output (cc/kg/day)	135	70	100	104	50	130	50
Urine output (cc/kg/day)	55	41	55	56.6	96	39	100
Salvage procedure day	45	73	59	176	125	47	128
Salvage procedure	Modified Kimura procedure	Stoma closure	Stoma closure	Fistula closure	Stoma closure	Decrease feeding/Stoma closure	Stoma closure

Six patients received ileostomy, and one patient had jejunostomy. Surprisingly, we found that all patients with high output stoma had increased aldosterone levels (Table 2). These patients were asymptomatic (cases 2 and 3), manifested dehydration with bodyweight loss or oliguria (cases 1, 5, and 6), or experienced cardiac events (cases 4 and 7) at the time we diagnosed them as PHA. Interestingly, only half of them had classical hyponatremia and hyperkalemia when abnormal high-level aldosterone and renin were detected. Abdominal ultrasonography and urinalysis revealed no renal abnormalities or urinary tract infections in all the cases. All patients accepted salvage operation, such as modified Kimura procedure (6), stoma closure, or fistula closure. Before the salvage procedure, we let the patients decrease oral intake or gave them St. Mark's solution. Electrolyte imbalance and high aldosterone level were corrected to normal range after the salvage procedure. Based on the above clinical description and blood test results, we diagnosed all the cases with secondary PHA following ileostomy and successfully corrected them by ileostomy closure. Long-term follow-up for these patients was healthy, except one patient who died from sepsis.

**Table 2:** Biochemical and hormonal profile of the patients.

**Case**	**1**	**2**	**3**	**4**	**5**	**6**	**7**
Onset time (Postnatal days)	30	62	39	89	5	38	62
Initial symptoms	Dehydration	None	None	Bradycardia	Dehydration	Dehydration	Cardiac arrest
	Body weight loss			Body weight loss	Body weight loss	Oliguria	
Na (mEq/L)	126	126	122	125	120	126	125
K (mEq/L)	6.12	5.31	6.85	6.8	3.4	5.1	4.4
Urine Na (mmol/L)	<10	37	26	14	57	N/A	<20
Aldosterone^*^ (ng/dL)	>1630	>1630	>1630	1471	372	346	383
Renin activity^#^ (ng/mL/hr)	N/A	>63	>63	N/A	N/A	31.9	N/A
pH	7.36	7.41	7.34	7.36	7.26	7.41	7.33
HCO3^−^ (mEq/L)	14.5	14	18	24	25.1	22.8	25.8
After salvage procedure					
Interval to following data	18 months	7 days	46 days	7 days	1 month	9 days	7 days
Aldosterone (ng/dL)	18.6	89.4	369	14.5	31	174	N/A
Renin activity (ng/mL/hr)	1.49	28.3	4.11	N/A	N/A	9.7	N/A
Na (mEq/L)	138	139	140	137	135	134	136
K (mEq/L)	3.76	4.49	5.44	4.7	3.6	4.9	4.0

* *Normal range of aldosterone: 2–70 ng/dL*.

# *Normal range of renin: in the premature infant is 11–167 ng/ml/h, while the range in infants is 1.4–7.8 ng/ml/h*.

## Discussion/Conclusion

PHA can be divided into three types, namely, PHA1, PHA2, and secondary PHA. PHA1 and PHA2 result from mutations for epithelial sodium channel (ENaC) subunits and renal outer medullary potassium (ROMK) channel, respectively (5, 7, 8). Infants with primary PHA present failure to thrive, anorexia, nausea, vomiting, hypotension, hyperkalemia, hyponatremia, and metabolic acidosis and are associated with high aldosterone and renin levels (9–11). Secondary PHA (but not primary PHA) was thought to be caused by transient aldosterone resistance.

Aldosterone is essential for sodium retention in the kidney, salivary glands, sweat glands, and colon. Transient or secondary PHA results from hydronephrosis and urinary tract infection in most cases, and nearly no gastrointestinal losses-related PHA was reported in infants (3, 4). In adults, several studies reported that secondary PHA developed in patients who underwent colectomy or small bowel resection (12–14). The colon and ileum are key sites for sodium and water absorption. Patients with ileostomy encounter problems of sodium loss if deprived of diet salt. Plasma renin activity and plasma aldosterone concentration were elevated in patients with dietary sodium deprivation for 1 day. Meanwhile, decreased sodium and increased potassium level in ileal effluent after salt deprivation suggest that the ileum is mediated by the renin–angiotensin–aldosterone system (13). In patients with high output ileostomy and jejunostomy, chronic plasma volume depletion and sodium loss were noted. In some severe cases, PHA may develop (15–17). Only a few case reports indicated patients with secondary PHA due to excessive gastrointestinal losses through ileostomy, and the condition resolved when the ileostomy was closed (17–20). In our case series, we examined serum aldosterone level in patients with high output stoma while patient had body weight loss or electrolyte imbalance. Surprisingly, we found high serum aldosterone and hyperkalemia instead of hypokalemia, which theoretically results from ileostomy potassium loss in most cases.

Moreover, the condition improved when we decreased feeding amount and gave intravenous fluid support. To prevent further stoma loss, related hyponatremia is the main key point for secondary PHA to develop. One study suggested the importance of preservation of part of the colon for maintenance of fluid and electrolyte balance in patients with extensive bowel resection (15).

The limitation of the study is that this case series has a variety of diagnoses, gestational ages, and body weights.

Due to the corrected PHA, we did not perform a genetic examination for PHA1 or PHA2. In addition, examination of renin and aldosterone was time-consuming and self-paid by family; it was tested depending on the clinical condition. Lack of data concerning renin blood levels was inevitable. Not all patients with PHA presented hyponatremia and hyperkalemia.

In conclusion, these cases we presented support PHA secondary to excessive gastrointestinal losses through high output stoma in the pediatric population. There are very few studies reporting this metabolic disorder in pediatric patients who undergo ostomy. However, this phenomenon is not rare in extreme preemies and newborns and deserves careful observation for the preterm infants who undergo ileostomy. It should be included in the differential diagnosis for children who present with salt-losing crises especially in a patient with ileostomy or jejunostomy. Once PHA is diagnosed, it may be corrected after stoma reversal. Additionally, we suggest immediately decreasing the feeding amount besides correcting electrolytes when PHA is suspected.

## Data Availability Statement

The data that support the findings of this study are available on request from the corresponding author. The data are not publicly available due to privacy or ethical restrictions. Written informed consent was obtained from the individual(s) and Ditmanson Medical Foundation Chia-Yi Christian Hospital for the publication of any potentially identifiable images or data included in this article.

## Ethics Statement

Written informed consent was obtained from the relevant individual(s), and/or minor(s)' legal guardian/next of kin, for the publication of any potentially identifiable images or data included in this article.

## Author Contributions

S-TC and C-YO: substantial contributions to the conception or design of the work. C-YO, G-BL, and M-FC: the acquisition, analysis, or interpretation of data for the work. C-YO, Y-JC, and S-TC: drafting the work or revising it critically for important intellectual content. All authors contributed to the article and approved the submitted version.

## Funding

This work was supported by grants from Ditmanson Medical Foundation Chia-Yi Christian Hospital (IRB2021006). The funders had no role in study design, data collection and analysis, and writing the manuscript.

## Conflict of Interest

The authors declare that the research was conducted in the absence of any commercial or financial relationships that could be construed as a potential conflict of interest.

## Publisher's Note

All claims expressed in this article are solely those of the authors and do not necessarily represent those of their affiliated organizations, or those of the publisher, the editors and the reviewers. Any product that may be evaluated in this article, or claim that may be made by its manufacturer, is not guaranteed or endorsed by the publisher.
